# Synthesis and Kinetic evaluation of an azido analogue of methylerythritol phosphate: a Novel Inhibitor of *E*. *coli* YgbP/IspD

**DOI:** 10.1038/s41598-018-35586-y

**Published:** 2018-12-17

**Authors:** Zoljargal Baatarkhuu, Philippe Chaignon, Franck Borel, Jean-Luc Ferrer, Alain Wagner, Myriam Seemann

**Affiliations:** 1 Laboratory of Biofunctional Chemistry, Faculté de Pharmacie - UMR 7199, Université de Strasbourg, 74 route du Rhin, 67401 Illkirch-Graffenstaden, France; 2 Laboratoire de Chimie Biologique et Applications Thérapeutiques, Institut de Chimie - UMR 7177, Université de Strasbourg, CNRS, 4 rue Blaise Pascal, 67070 Strasbourg, France; 3 Institut de Biologie Structurale IBS, Université Grenoble Alpes, CEA, CNRS, 38044 Grenoble, France

## Abstract

As multidrug resistant pathogenic microorganisms are a serious health menace, it is crucial to continuously develop novel medicines in order to overcome the emerging resistance. The methylerythritol phosphate pathway (MEP) is an ideal target for antimicrobial development as it is absent in humans but present in most bacteria and in the parasite *Plasmodium falciparum*. Here, we report the synthesis and the steady-state kinetics of a novel potent inhibitor (MEPN_3_) of *Escherichia coli* YgbP/IspD, the third enzyme of the MEP pathway. MEPN_3_ inhibits *E*. *coli* YgbP/IspD in mixed type mode regarding both substrates. Interestingly, MEPN_3_ shows the highest inhibitory activity when compared to known inhibitors of *E*. *coli* YgbP/IspD. The mechanism of this enzyme was also studied by steady-state kinetic analysis and it was found that the substrates add to the enzyme in sequential manner.

## Introduction

Drug resistance is an ever-growing concern that poses a major challenge for new drug development. In the field of antibiotic discovery, the situation is alarming as some infections are already impossible to treat due to resistance. *Enterobacteriaceae*, in particular *Klebsiella pneumonaiae* and *Escherichia coli*, are highly pervasive in community-acquired and nosocomial infections. The worldwide emergence of carbapenemase-producing *Enterobacteriaceae* represents a serious public health threat as carbapenems are often the last option for treatment of patients infected by these bacteria^1^. WHO already estimated in 2014 that 44% of its member states reported *E*. *coli* strains resistant to third-generation cephalosporins and fluoroquinolones and highlighted the high resistance rates of *E*. *coli* strains to the last-generation drugs^2^. In September 2017, WHO classified carbapenem-resistant and third-generation cephalosporin resistant *Enterobacteriaceae* among the most critical priority for Research and Development of new antibiotics as strains that cannot be fought by any antibiotic on the market are emerging worldwide^3^. Given the severe threat from organisms resistant to conventional antibacterial agents, targeting the MEP pathway responsible for the biosynthesis of the universal isoprenoid precursors in most bacteria and in the parasite responsible for malaria was proposed as an attractive strategy in the search for new antimicrobial agents^4–7^.

**Figure 1:**
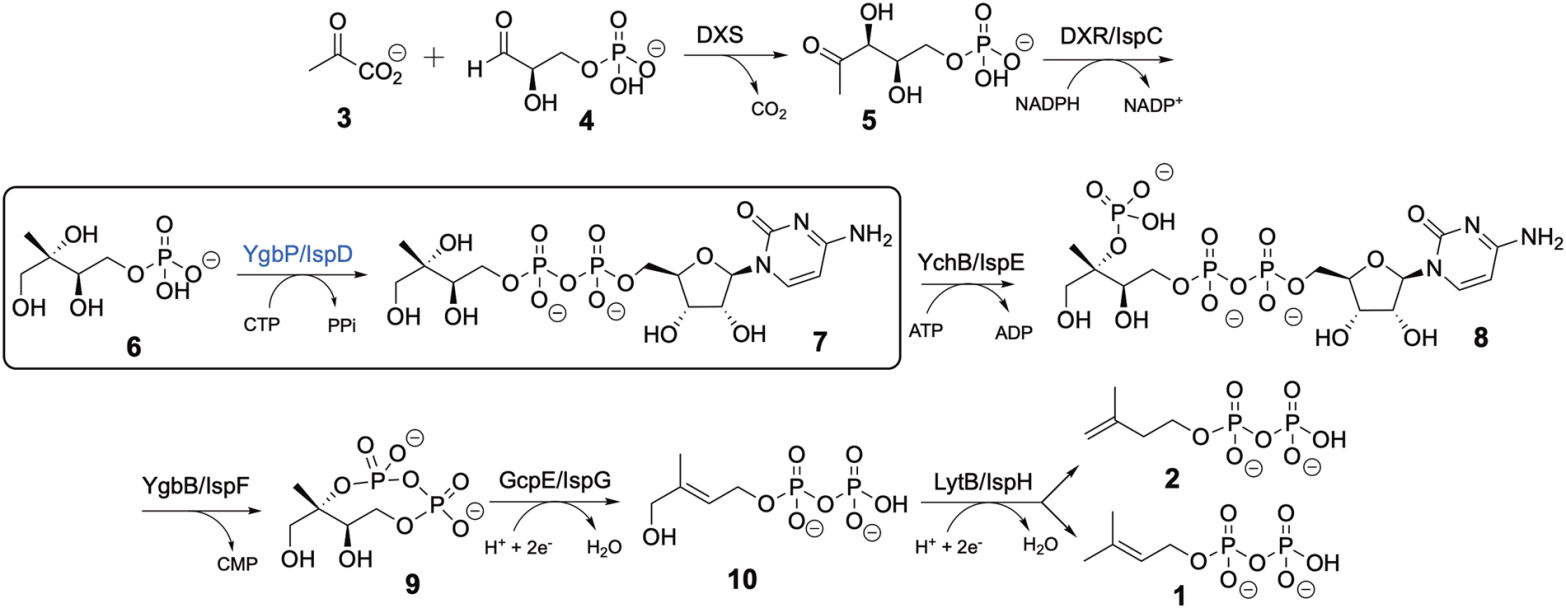
MEP pathway.

Isoprenoids are the most diverse family of natural products that comprises over 55000 known compounds. They are found in all living organisms and are involved in numerous essential biological processes such as electron transport, cell-wall biosynthesis, and protein prenylation^8,9^. Isoprenoids are synthesised through multiple condensation of two main building blocks: dimethylallyl diphosphate (DMADP, **1**) and isopentenyl diphosphate (IDP, **2**, Fig. 1)^8^. The MEP pathway, absent in humans, is an alternative to the well-known mevalonate pathway existing in animals^10^ for the formation of IDP and DMADP. The MEP pathway (Fig. 1) starts with condensation of pyruvate (**3**) and glyceraldehyde 3-phosphate (**4**) to form 1-deoxy-D-xylulose 5-phosphate (**5**), which is further converted to 2-*C*-methyl-D-erythritol 4-phosphate (MEP, **6**). MEP reacts with cytidine triphosphate (CTP) to generate 4-diphosphocytidyl-2-*C*-methyl-D-erythritol (CDP-ME, **7**), which is further phosphorylated to yield 4-diphosphocytidyl-2-*C-*methyl-D-erythritol 2-phosphate (CDP-MEP, **8**). After cyclisation and cytidine monophosphate (CMP) release, 2-*C*-methyl-D-erythritol 2,4-cyclodiphosphate (ME-cPP, **9**) forms. ME-cPP then generates (*E*)-4-hydroxy-3-methylbut-2-en-1-yl diphosphate (HMBDP, **10**) that further produces DMADP and IDP.

**Figure 2:**
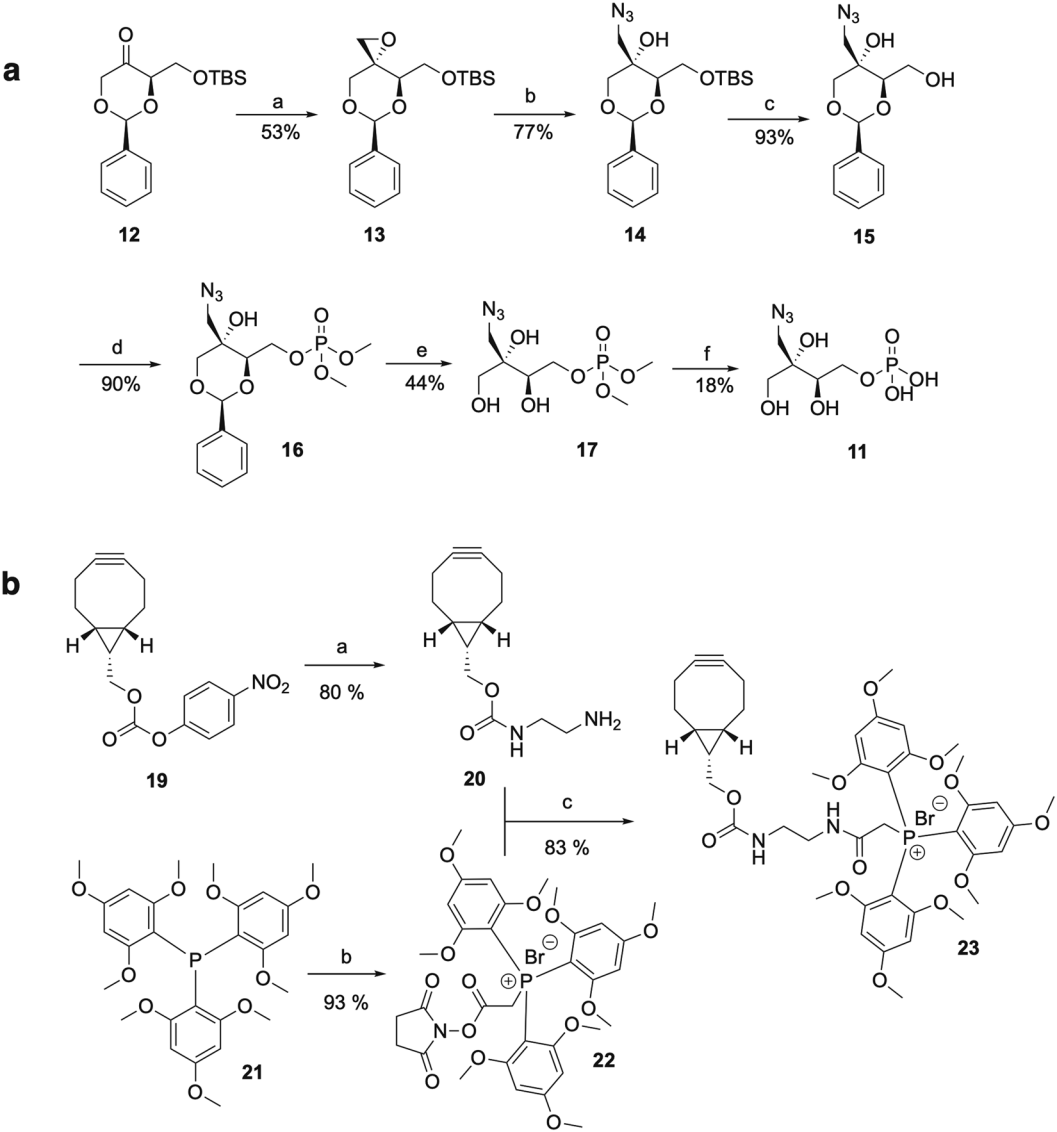
Synthesis of MEPN_3_ (**11**) and BCN-TMBPP (**23**). (**a**) Synthetic route to MEPN_3_. (**a**) (CH_3_)_3_SOI, NaH, DMSO, 10 min, r.t; (**b**) NaN_3_, NH_4_Cl, DMF, 2 h, 60 °C; (**c**) TBAF, THF, 0 °C to r.t; (**d**) (CH_3_O)_2_POCl, DMAP, DCM, 2 h, 0 °C; (**e**) DOWEX (H^+^), MeOH, 48 h, r.t; (**f**) (CH_3_)_3_SiBr, H_2_O, DCM, 0 °C to r.t. (**b**) Synthetic scheme for BCN-TMPP. (**a**) Ethylenediamine, TEA, DMF, 2 h, r.t; (**b**) Bromoacetic acid *N*-hydroxysuccinimide ester, toluene, 0.5 h, r.t; (**c**) NEt_3_, DCM, 16 h, r.t.

To date only one compound targeting the MEP pathway, namely fosmidomycin, an inhibitor of the second enzyme 1-deoxyxylulose 5-phosphate reductoisomerase (DXR), is under clinical trial as an antimalarial agent in combination with clindamycin and piperaquine^11^, highlighting the potential of the MEP pathway for drug development^12^. Exploring new inhibitors of the MEP pathway could be a source of new therapeutic agents that are urgently needed to fight life-threatening infections. Here we report the synthesis and inhibition studies of MEPN_3_, (**11**, Fig. 2a) as a potential inhibitor of *E*. *coli* YgbP (also called IspD), the third enzyme of the MEP pathway.

## Results and Discussion

### *E*. *coli* YgbP/IspD

*E*. *coli* YgbP/IspD (EC 2.7.7.60) is encoded by the *ygbP* gene and catalyses the transformation of MEP and CTP into 4-diphosphocytidyl-2-C-methyl-D-erythritol (CDP-ME; **7**) and inorganic diphosphate (PPi) (Fig. 1) in the presence of a divalent cation such as Mn^2+^, Mg^2+^ or Co^2+^^13^. YgbP was checked for activity using a similar method as described previously and based on the transformation of inorganic diphosphate to phosphate by inorganic pyrophosphatase followed by the quantification of the resulting phosphate by complexation with malachite green ammonium molybdate^14^. This method is robust, simple, fast, reliable and inexpensive for checking the activity of YgbP. The activity of YgbP limiting the concentration of CTP to 200 µM (as substrate inhibition was reported at high concentrations^15^) was 3.47 µmol.min^−1^.mg^−1^ and is in the same range as published^14^. The kinetic parameters of YgbP/IspD were determined using either varied MEP concentrations and a fixed CTP concentration (200 µM) or varied CTP concentrations and a fixed MEP concentration (250 µM). The reaction rates could be fitted according to the Michaelis–Menten equation and apparent *K*_*m*_ values of 40 ± 7 µM for MEP (*k*_*cat*_ = 1.77 s^−1^) and 84 ± 9 µM for CTP (*k*_*cat*_ = 6.87 s^−1^) were estimated, which are in agreement with the reported values using the same method for the quantification of the activity^14^. A similar *K*_*m*_ for MEP (32 ± 3 µM) was reported by Cane *et al*.^16^ using a radiolabeled assay. Rohdich *et al*.^13^ published a smaller *K*_*m*_ for MEP (3.14 µM) but a similar *K*_*m*_ for CTP (131 µM) when the detection of inorganic diphosphate was achieved indirectly after its consumption through a cascade of reactions leading to the reduction of NADP^+^. Richard *et al*.^15^ reported 370 ± 60 µM (10-fold higher than our result) *K*_*m*_ value for MEP but used different conditions with high CTP concentrations (above 7 mM).

Our aim was to design an *E*. *coli* YgbP/IspD inhibitor that would be suitable for fragment-based drug discovery approaches. We searched a position for the insertion of an azide functionality while introducing minimal structural perturbation on the MEP substrate and keeping chemical stability. Therefore, we tested MEPN_3_ (**11**, Fig. 2), a MEP analogue harbouring an azido on the methyl group.

### Synthesis of MEPN_3_

The route to MEPN_3,_ (**11**) is outlined in Fig. 2 and starts with ketone **12** which was previously described by Coates and coworkers^17^. Submitted to a Corey–Chaykovsky reaction, ketone **12** was diastereoselectively converted to epoxide **13**^18,19^; this represents a valuable improvement compared to Coates’ synthesis of **13**, which featured a two-step “olefin formation-epoxidation” procedure that led to a diastereomeric mixture^17^. Epoxide **13** was further transformed into azido alcohol **14** with sodium azide, followed by deprotection of the primary alcohol with tetra-*n*-butylammonium fluoride. The resulting azido diol **15** was phosphorylated with dimethyl chlorophosphate to yield compound **16**. Hydrolysis of benzylidene acetal with an acidic resin followed by deprotection of the phosphate group using a McKenna reaction^20^ afforded MEPN_3_ (**11**).

### MEPN_3_ is a poor substrate of YgbP/IspD

As MEPN_3_ (**11**) structurally resembles MEP, YgbP was further assayed using MEPN_3_ as a substrate. Poulter *et al*.^21^ have previously reported that 2-*C*-ethyl-D-erythritol phosphate was a substrate for *Agrobacterium tumefaciens* YgpP/IspD showing replacement of the methyl at C-2 of MEP by an alternative substituent could still allow catalysis. Initial studies showed that MEPN_3_ was a substrate of YgbP but the enzymatic reaction velocity declined at higher MEPN_3_ concentrations (>500 µM), revealing substrate inhibition (Fig. S1). From the obtained data, the activity of YgbP (at 300 µM of MEPN_3_) was 100-fold less than the activity of YgbP with MEP (at 250 µM) showing that MEPN_3_ is a poor substrate.

**Figure 3:**
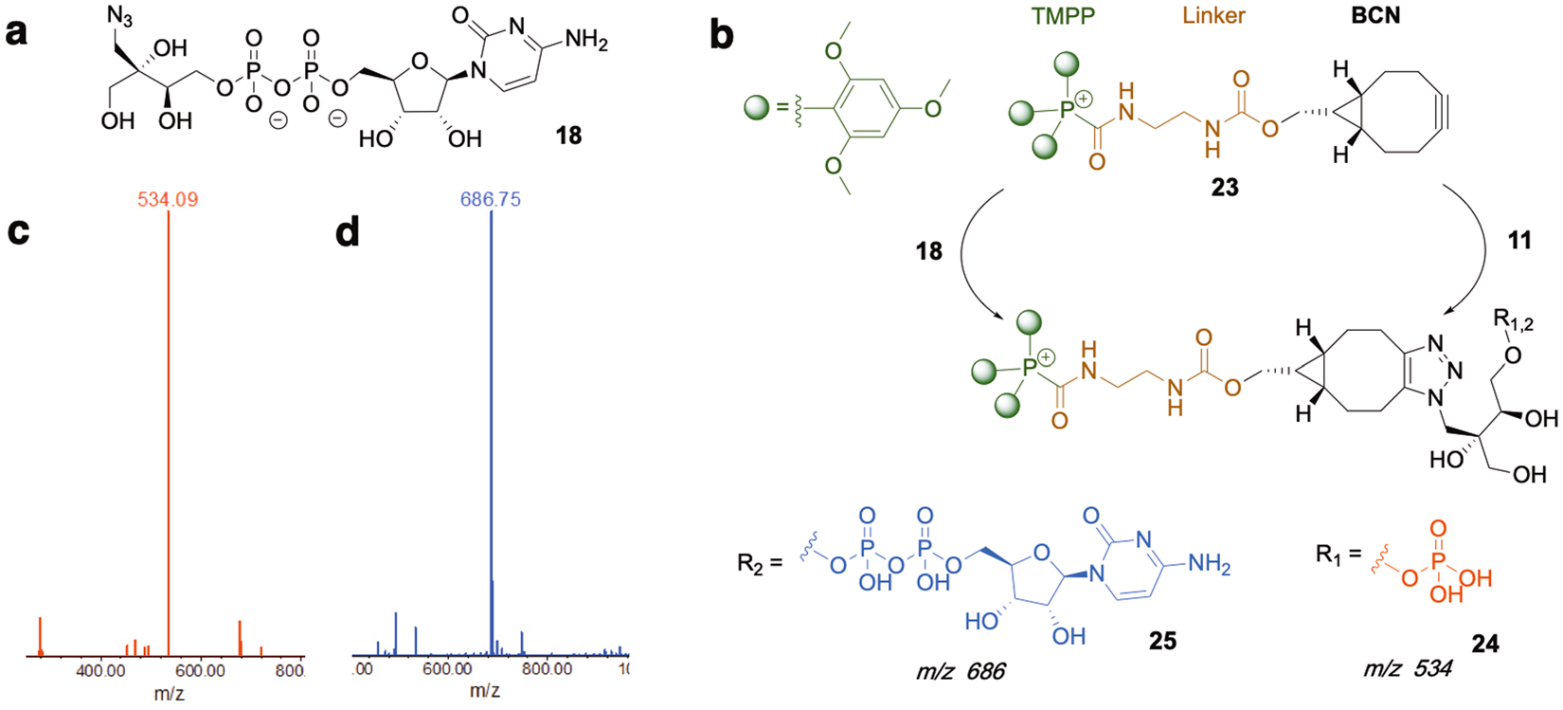
LC-MS analysis of MEPN_3_ or its product in the YgbP catalysed reaction after derivatisation with TMPP-BCN (**23**). (**a**) Structure of CDP-MEN_3_ (**b**) Structure of MEPN_3_ or of its product after derivatisation with TMBPP-BCN. (**c**) Mass-to-charge ratio of MEPN_3_ clicked with TMPP-BCN. (**d**) Mass-to-charge ratio of the product of **11** in the YgbP catalysed reaction clicked with **23**.

The previous assay was based on the detection of the released diphosphate but not on the detection of CDP-MEN_3_ (**18**) (Fig. 3a) that should be produced if MEPN_3_ behaved like MEP in the active site of YgbP. In this context, strain-promoted alkyne-azide cycloaddition (SPAAC) using BCN ((1*R*,8*S*,9*S*)-bicyclo[6.1.0]non-4-yn-9-yl)methanol) derivative **23** encompassing a TMPP (tris(2,4,6-trimethoxyphenyl phosphonium, **21**, Fig. 2) tag was employed to detect CDP-MEN_3_ (**18**) in the YgbP assay with MEPN_3_ as substrate. TMPP had been previously applied as a charge derivatisation agent for small biological molecules to enhance their detectability using positive ion ESI-MS analysis, as it carries a permanent positive charge^22–24^. BCN-TMPP (**23**) was prepared as described in Fig. 2. BCN was activated with *p*-nitrochloroformate to generate molecule **19**^25^ which was further converted into compound **20** using ethylenediamine^26^. In parallel, TMPP and bromoacetic acid *N*-hydroxysuccinimide ester were used to produce TMPP derivative **22**. Reaction of BCN derivative **20** with the activated TMPP (**22**) afforded the target molecule **23** in good yield.

In a preliminary experiment, BCN-TMPP was incubated with MEPN_3_ and after LC-MS analysis (C_18_ column, UV detection for LC, positive mode detection for MS) the major product was detected at *m/z* 534 Da (calculated mass = 1067 Da) consisting of BCN-TMPP-MEPN_3_ (**24**) (Fig. 3b) carrying a second positive charge in addition to TMPP’s permanent charge (Fig. 3c). In order to identify the product of YgbP using MEPN_3_ as substrate, the completed enzymatic reaction was incubated with BCN-TMPP, and after LC-MS analysis, a product displaying *m/z* 686 Da corresponding to **25** (calculated mass = 1372 Da) harboring two charges was detected confirming that MEPN_3_ was turned over by YgbP to CDP-MEN_3_ (Fig. 3d). The fact that MEPN_3_ was a poor substrate compared to MEP was surprising as the only difference between both molecules was the azide function. To better understand the slow turnover of MEPN_3_ by YgbP, we further evaluated the inhibition potential of **11** on YgbP.

### Inhibition of *E*. *coli* YgbP/IspD by MEPN_3_

**Figure 4:**
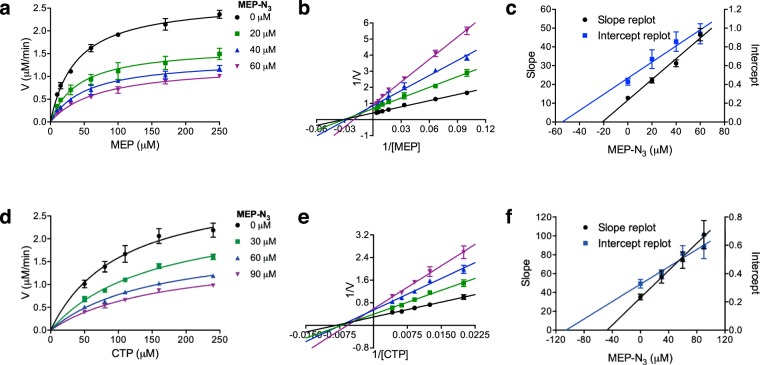
Inhibition of *E*. *coli* YgbP by MEPN_3_. (**a**) Steady state kinetics at variable MEP concentrations and fixed MEPN_3_ concentrations (0 µM, r^2^ = 0.99; 20 µM, r^2^ = 0.98; 40 µM, r^2^ = 0.99; 60 µM, r^2^ = 0.99). (**b**) Double reciprocal plot of (**a**) (0 µM, r^2^ = 0.99; 20 µM, r^2^ = 0.99; 40 µM, r^2^ = 0.98; 60 µM, r^2^ = 0.99) (**c**) Replot of slope and intercept of (**b**) (slope, r^2^ = 0.98; intercept, r^2^ = 0.95). (**d**) Steady state kinetics at variable CTP concentrations and fixed MEPN_3_ concentrations (0 µM, r^2^ = 0.98; 30 µM, r^2^ = 0.99; 60 µM, r^2^ = 0.99; 90 µM, r^2^ = 0.98). (**e**) Double reciprocal plot of (**d**) (0 µM, r^2^ = 0.99; 30 µM, r^2^ = 0.98; 60 µM, r^2^ = 0.98; 90 µM, r^2^ = 0.99). (**f**) Replot of slope and intercept of (**e**) (slope, r^2^ = 0.99; intercept, r^2^ = 0.95). Mean and SEM values are displayed, n ≥ 3.

Preliminary experiments revealed the YgbP reaction rate using its natural substrates (MEP and CTP) was decreasing when MEPN_3_ was present. In order to further determine the inhibition parameters and the inhibition mode of MEPN_3_, steady-state inhibition kinetic studies were performed (Fig. 4, a,d). The data were fitted according to the double reciprocal analysis (Fig. 4b,e) and highlighted that MEPN_3_ inhibited YgbP in a mixed type inhibition mode with respect to both substrates. From the replots (Fig. 4c,f), we have found *K*_*i*_ = 21 ± 3 µM and α*K*_*i*_ = 54 ± 4 µM (α = 2.5) when MEP was the varied substrate while *K*_*i*_ = 47 ± 6 µM and α*K*_*i*_ = 105 ± 24 µM (α = 2.2) when CTP was the varied substrate. As α*K*_*i*_ value was higher than *K*_*i*_ value in both cases, it suggests that MEPN_3_ has a higher affinity for the free enzyme than for the enzyme–substrate complex.

**Figure 5:**
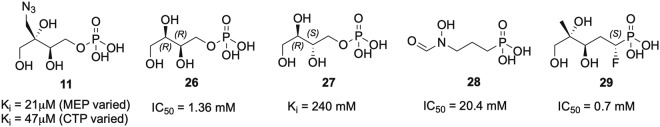
Structures of *E*. *coli* YgbP described (**26–28**) and new (**11**) inhibitors. Ki values are displayed when available, otherwise they are replaced by the reported IC_50_ values.

Although there are number of reports describing potent inhibitors of YgbP from malaria parasites^27,28^ and *Mycobacteria*^29,30^ as well as from the plant *Arabidopsis thaliana*^31–33^, there are hardly any inhibitors reported for *E*. *coli* YgbP (Fig. 5) and they all display very high IC_50_ reflecting their poor inhibition potential. The first inhibitor described for *E*. *coli* YgbP was D-erythrol-4-phosphate (**26**) with IC_50_ value of 1.36 mM which was also a substrate and reduced the turnover rate compared to natural substrate MEP^34^. Two years later, L-erythrol-4-phosphate (**27**) was reported to be a weak competitive inhibitor of MEP in *E*. *coli* YgbP, displaying a *K*_*i*_ value of 240 mM^35^. Interestingly fosmidomycin (**28**), the only inhibitor of MEP pathway currently in clinical trial and targeting DXR, was also reported to be an inhibitor of YgbP. Odom and co-workers reported an increase of MEP level and decrease of CDP-ME level when *Plasmodium falciparum* was treated with fosmidomycin and further showed that **28** inhibited *E*. *coli* YgbP activity with an IC_50_ value of 20.4 mM^36^. Interestingly, in the course of this work, Freel Meyers and co-workers^37^ described (*5S*)-D-methylerythritol monofluoromethyl phosphonate (**29**) as a new inhibitor of *E*. *coli* YgbP displaying an IC_50_ value of 0.7 mM. Even though no detailed kinetics were reported, these results encouraged us in our approach. MEPN_3_ is a mixed type inhibitor of *E*. *coli* YgbP regarding both substrates with *K*_*i*_ values in the micromolar range (21 µM and 47 µM). IC_50_ for MEPN_3_ was estimated to be around 41.5 µM (see Fig. S4), making MEPN_3_ the best inhibitor of *E. coli* YgbP known to date.

As MEPN_3_ is a substrate analogue of MEP, we expected the inhibition to be competitive with MEP and uncompetitive with CTP. In order to further understand the mixed type inhibition observed for MEPN_3_ on YgbP regarding both substrates, set of docking studies were performed to investigate the binding mode of **11**.

### Docking experiments of MEPN_3_ with *E*. *coli* YgbP

**Figure 6:**
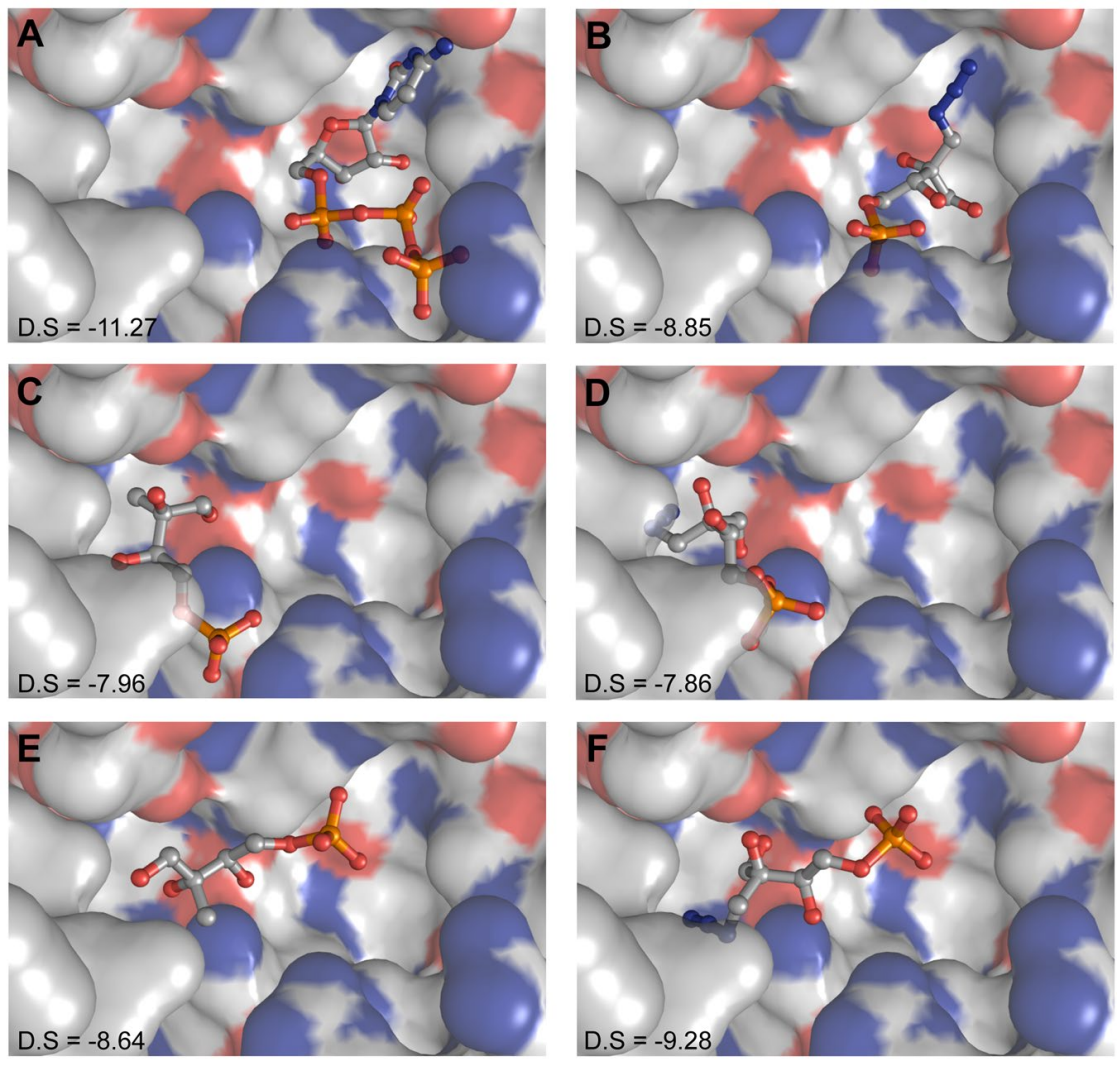
*In silico* docking results. Docking experiments were performed with the X-ray structure of *E*. *coli* YgbP: CTP complex (PDB ID: 1I52). The best docking poses and their corresponding docking scores (D.S) are reported. (**A**,**B**) CTP and respectively MEPN_3_ docked using a target with an empty binding pocket. (**C**) to (**F**) docking poses revealing the two possible binding sites observed for MEP (**C**,**E**) or MEPN_3_ (**D**,**F**) when the docking experiments were performed with a target already containing bound CTP. The compounds were docked using Glide in extra precision (XP) mode and the Glide docking score was used to rank the docking poses.

Three different X-ray structures of *E*. *coli* YgbP have been reported^38^: the apo form of the protein (1INJ) and its complexes with CTP (1I52) or CDP-ME (1INI). However, no structure of *E*. *coli* YgbP in complex with MEP has been obtained to date. To identify the mode of binding of **11**, we attempted to solve the crystal structure of *E*. *coli* YgbP in complex with **11** but we were unsuccessful. The lack of crystal structure of YgbP in complex with MEP or with its structural analogue MEPN_3_ may be due to an ordered sequential mechanism, in which CTP binds first to the enzyme followed by MEP, as proposed by Cane and co-workers^15^. To further investigate the binding mode of MEPN_3_, docking experiments were performed using the *E*. *coli* YgbP homodimeric CTP form (1I52). Indeed, this form appears to be the most suitable since it has the highest resolution and is the only one that displays a well-defined P-loop (residues 17–25)^38^. Two sets of docking were done: the first one using a protein target containing a bound CTP and a second one using an empty protein target. To avoid bias linked to the use of a too small docking area, we defined, for both cases, a search area covering the entire YgbP CTP/CDP-ME binding site. To validate our docking procedure, CTP for which the crystallographic structure in complex with YgbP is available, was submitted to our docking protocol. The docked CTP superimposed very well onto the CTP observed in the crystal structure. The closest docking pose displays a docking score of −11.27 and a RMSD value of 0.11 Å (Fig. 6).

When using a target already containing CTP, we were able to identify two binding sites for MEP and MEPN_3_ (Fig. 6): one deeply buried at the bottom of the CDP-ME pocket with docking scores of −7.86 and −7.96 for MEPN_3_ and MEP respectively, and a second site located closest to the surface of the protein which appears to be more favorable for the binding of the two molecules given the slightly better docking scores obtained in that case (−9.28 and −8.64 for MEPN_3_ and MEP respectively). When the empty protein (no CTP bound) was used as a target, we observed that MEPN_3_ preferentially docked into the CTP binding pocket with a docking score for the best pose of −8.85.

The docking results confirmed that MEPN_3_ can bind either to the CTP binding pocket or to the hypothetical MEP binding pocket explaining the mixed type inhibition of MEPN_3_ for *E*. *coli* YgbP revealed by the kinetic data. These results show that the very high inhibition potential of MEPN_3_ compared to the other *E*. *coli* YgbP described inhibitors is most probably due to the fact that MEPN_3_ can bind to both substrate pockets. The design of such inhibitors has never been achieved previously. Interestingly, fosmidomycin (**28**), has also been reported to bind to the CTP binding site of *E*. *coli* YgbP according to docking experiments^36^. The high potential of fosmidomycin as a drug compared to the other known DXR inhibitors might be linked to this additional binding property. The druggability of the CTP-binding pocket in the homologue protein of *M*. *tuberculosis* has actually been reported by Hirsch and co-workers^39^. In this context, the binding of MEPN_3_ into the CTP-binding site should be considered as a starting point for new antibacterial development.

As MEPN_3_ is an analogue of MEP, we further checked whether MEP could also bind to the CTP pocket of *E*. *coli* YgbP. Using the target without bound CTP, our experiments revealed that MEP also preferentially docks to this pocket with a docking score of −9.40 (Fig. S5). No other evidence suggesting the binding of MEP to the CTP-binding pocket has been reported but if this hypothesis based on docking were to be true, the multiple binding sites revealed here for MEP would be puzzling from the catalytic point of view. This has prompted us to further investigate the mechanism of *E*. *coli* YgbP using a more detailed kinetic analysis.

### Investigation of *E*. *coli* YgbP mechanism by using a bi-substrate kinetic analysis

**Figure 7:**
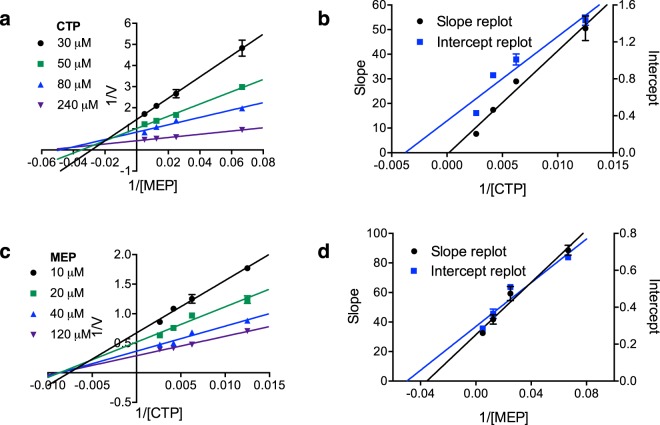
Bi-substrate steady-state kinetic analysis of *E*. *coli* YgbP. (**a**) Double reciprocal plot of initial velocities at variable MEP concentrations and fixed CTP concentrations (30 µM, r^2^ = 0.99; 50 µM, r^2^ = 0.99; 80 µM, r^2^ = 0.96; 240 µM, r^2^ = 0.99). (**b**) Slope and intercept replot of (**a**) (slope, r^2^ = 0.97; intercept, r^2^ = 0.90). (**c**) Double reciprocal plot of initial velocities at variable CTP concentrations and fixed MEP concentrations (10 µM, r^2^ = 0.99; 20 µM, r^2^ = 0.96; 40 µM, r^2^ = 0.95; 120 µM, r^2^ = 0.98). (**d**) Slope and intercept replot of (**c**) (slope, r^2^ = 0.97; intercept, r^2^ = 0.92). Mean and SEM values are displayed, n ≥ 3.

Cane and co-workers^15^ highlighted, using pulse-chase experiments, that the formation of CDP-ME was accomplished by an ordered sequential mechanism, in which CTP binds first to the enzyme followed by MEP binding. Nucleophilic attack on α-phosphate group of CTP by the phosphate moiety of MEP will then afford a pentacoordinate intermediate that will subsequently collapse to produce CDP-ME and diphosphate^15,38^. However, the ordered sequential catalytic mechanism of YgbP has never been characterised using a complete bisubstrate kinetic analysis. Such a kinetic analysis was performed here by measuring the velocity of different assays in which one of the substrate concentration was varied at different but fixed concentrations of the second substrate. Double reciprocal plots of initial velocity for both substrates resulted in lines intersecting left to the vertical axis and above to the horizontal axis (Fig. 7a,c) confirming that YgbP mechanism is sequential where ternary complex forms before any product release^40,41^.

**Figure 8:**
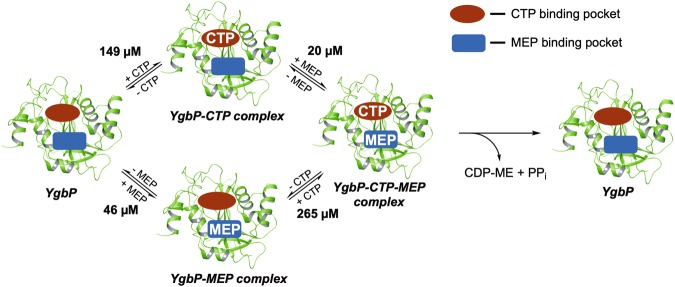
*E*. *coli* YgbP bi-substrate steady state kinetic analysis. For reasons of clarity, the scheme was simplified as in the YgbP-MEP complex, MEP could also bind in the CTP binding site. See Fig. S3 for detailed reaction mechanism.

After fitting the data to the rate equation for sequential bi-substrate mechanism (See supporting information for more details), the complete kinetic scheme for *E*. *coli* YgbP was obtained for the first time (Fig. 8). *K*_*m*_ values of 149 μM (*K*_*iA*_) for CTP and 46 μM (*K*_*iB*_) for MEP were retrieved and were found to be in agreement with the *K*_*m*_ values obtained using the classical Michaelis–Menten equations (*K*_*m*_ = 84 μM for CTP and *K*_*m*_ = 40 μM for MEP). The dissociation constant of YgbP-CTP complex for MEP (*K*_*B*_) was revealed to be very low (20 μM) while the dissociation constant of YgbP-MEP complex for CTP (*K*_*A*_) was thirteen times higher (265 μM). These values show that if CTP binds to the free enzyme first, the affinity of MEP for YgbP-CTP complex is increased (low dissociation constant) leading to the production of CDP-ME. If MEP binds to the free enzyme first, the affinity of CTP for YgbP-MEP complex will be very low (high dissociation constant) and the YgbP-MEP complex might not be productive and would dissociate to regenerate the free enzyme. This achievement is compatible with the hypothesis that MEP could bind to the CTP pocket and if this happens, the resulting complex would be expected to dissociate to allow the binding of CTP in the CTP pocket. Then, once MEP is present in the active site, the reaction would proceed.

### Selectivity of MEPN_3_

Fosmydomycin is the only inhibitor of the MEP pathway currently under clinical trial. It targets DXR but also to a weaker extend YgbP. This double targeting of fosmidomycin for the MEP pathway is very interesting and may be at the origin of the high potential of fosmidomycin as an antibiotic compared to the other MEP pathway inhibitors. This unique property of fosmidomycin encouraged us to investigated whether MEPN_3_ is selective for YgbP or if it targets another enzyme of the MEP pathway. In this context, DXR was investigated as a potential target for our inhibitor, as it lays just before YgbP in the MEP pathway and produces MEP. Docking experiments were carried out based on the structure of *E*. *coli* YgbP in complex with 1-deoxy-D-xylulose 5-phosphate (**5**) and NADPH (1Q0Q). Two sets of docking were performed: one with only NADPH bound and another with the empty target. Interestingly, we observed that MEPN_3_ binds in the binding site of the substrate **5** with a docking score of −9.09 for the best pose (Fig. S6). These results need to be further confirmed using kinetic investigations on DXR but they already highlight that MEPN_3_ is a good starting point in the search for new drugs as it might target several enzymes of the MEP pathway.

## Conclusion

We have successfully synthesised MEPN_3_, the best inhibitor of *E*. *coli* YgbP/IspD known to date and the first inhibitor shown to bind to either or both substrate binding sites. This special binding feature appears to be at the origin of the potency of MEPN_3_. In addition, our in-depth kinetic studies of YgbP, using a bi-substrate model for the first time also highlighted that the binding of MEP to the free enzyme disfavored the formation of the product. Building on the knowledge gained from our study, new inhibitors derived from MEPN_3_ might be further elaborated either by developing new analogs bearing this dual binding profile or via structure-based fragment selection and *in situ* chemistry since MEPN_3_ is already a good starting point for such strategy. With this aim, fragments could be among other MEP analogues or other molecules binding to the CTP pocket. In this context, preliminary docking experiments were performed using the empty target and a ligand obtained by replacing the azido function of **11** by a methyltriazole moiety. Docking scores and the pose obtained when **11** is in the CTP pocket show that fragment growing using click chemistry is feasible.

Therefore, the discovery of MEPN_3_ as a new YgbP inhibitor as well as its unusual mode of action paves the way for original approaches toward the discovery of drug candidates that are urgently needed for the treatment of antimicrobial-resistant *Enterobacteriacea* infections.

## Materials and Methods

### General conditions for enzyme kinetics

Colorimetric assay reported by Bernal *et al*.^14^ was used with some modifications. The standard reaction mixture contained 50 mM Tris-HCl pH = 8, 1 mM MgCl_2_, 1 mM DTT, 133 mU/mL of inorganic pyrophosphatase, 200 µM CTP (when MEP was the variable substrate), 250 µM MEP (when CTP was the variable substrate) unless otherwise stated and 0.065 µg YgbP enzyme in the final volume of 400 µL. Assays were initiated by addition of YgbP and incubated eight min at 30 °C before being quenched with 100 µL dye reagent (for preparation see SI). The assays were further incubated for ten min before measuring OD at 630 nm. A blank reaction that contained every component except YgbP was carried out at the same time for each assay and the corresponding OD_630_ value of the blank was subtracted from the OD_630_ value measured for the assay. The phosphate concentration of the assays was determined from standard curves obtained by measuring OD_630_ values of different phosphate standards with concentrations varying from 2 to 30 µM. Data were fitted with the least-squares method to the corresponding equations using GraphPad Prism 7.

### YgbP kinetic parameter determination

MEP concentrations were 15, 30, 45, 60, 100, 150 and 250 µM when MEP was the variable substrate and CTP concentration was fixed at 200 µM. CTP concentrations were 30, 60, 90, 120, 180, 270 and 450 µM when CTP was the variable substrate and MEP concentration was fixed at 250 µM.

### YgbP inhibition kinetic studies

Steady-state kinetic constants were determined from different assays at several fixed inhibitor concentrations and varying the concentration of one substrate and keeping the concentration of the other substrate constant. The initial velocities and concentrations were fitted according to the appropriate model of inhibition^42^.

### YgbP mechanism determination

YgbP bi-substrate kinetic assays were performed, first by varying MEP concentrations at several fixed CTP concentrations and second by varying CTP concentrations at several fixed MEP concentrations. The data were fitted to the corresponding equations to determine kinetic values as described in SI.

### YgbP kinetic studies with MEPN_3_ as a substrate

MEPN_3_ concentrations were 40, 150, 300, 400, 600, 1000, 1500, 2000 and 3000 µM and CTP concentration was fixed at 200 µM.

### MEPN_3_ as substrate of YgbP

MEPN_3_ (0.2 mM) was added to a mixture of CTP (1 mM), MgCl_2_ (5 mM), DTT (1 mM) in a final volume of 200 μL buffer (Tris HCl, 50 mM pH = 8). *E*. *coli* YgbP (34 µg) was added to initiate the reaction. The reaction mixture was incubated at 30 °C for one h then MeCN (200 µL) was added, and the mixture was left at 0 °C for 20 min to precipitate proteins. The precipitate was removed by centrifugation (13000 rpm, 10 min). BCN-TMPP (0.8 mM) was added to the supernatant that was further incubated at 37 °C overnight. The mixture was analysed by LC-MS (Waters Alliance 2690 LC system with C_18_ column coupled with Waters ACQUITY QDa mass detector) using 10 µL of injection volume.

### Docking experiments

*In silico* docking experiments were carried out with the Schrödinger suite (Schrödinger LLC, New York, NY, USA). The X-ray structure of the CDP-ME synthase with CTP (PDB ID 1I52) was used for the studies^38^. The protein structure was processed with the protein preparation wizard tool. Ligand 3D structures, tautomers and ionisation states were produced with LigPrep. The CDP-ME binding pocket was used to generate the docking grid. We defined a docking area of 32 Å × 32 Å × 32 Å centered on the reaction product (CDP-ME). No constraints (such as hydrogen bond or atom position) were applied to guide the binding. All the compounds were docked using Glide^43^ in extra precision (XP) mode^44^. The Glide docking score was used to rank the docking poses.

## Electronic supplementary material

Supporting information
