# Supplementary material for: Synthesis and Kinetic evaluation of an azido analogue of methylerythritol phosphate: a Novel Inhibitor of E. coli YgbP/IspD
Source: Sci Rep. 2018 Dec 17;8:17892. doi: 10.1038/s41598-018-35586-y (PMC6297244; doi:10.1038/s41598-018-35586-y)
Supplement: Supplementary file 1 — Supporting information [file 41598_2018_35586_MOESM1_ESM.pdf]

Supporting Information

Synthesis and Kinetic evaluation of an azido analogue of methylerythritol phosphate: a Novel Inhibitor of *E. coli* YgbP/IspD.

Zoljargal Baatarkhuu, Philippe Chaignon, Franck Borel, Jean-Luc Ferrer, Alain Wagner and Myriam Seemann

General information.

All chemicals and solvents were purchased from commercial suppliers. Reactions were performed under N_2 atmosphere unless otherwise stated. TLC was performed on Merck TLC Silica gel 60 F_{254} aluminum sheets and revealed under 254 nm UV light, or with stains (vanillin or $KMnO_4$). NMR spectra were recorded on Brucker Avance 400 and 500 MHz spectrometers. Chemical shifts (δ) are represented in parts per million (ppm). Residual non-deuterated solvent were used for ^{1}H-NMR calibration ($CDCl_3$ δ = 7.26 ppm, CD_3OD δ = 3.31 ppm, D_2O δ = 4.79 ppm). Residual solvent peaks from $CDCl_3$ δ = 77.16 ppm and CD_3OD δ = 49 ppm were used as a reference in ^{13}C-NMR. External calibration by 85 % H_3PO_4 (- 0.85 ppm) was used for referencing of ^{31}P-NMR. IR spectra were recorded on Nicolet 380 FT-IR spectrometer. UV visible measurements were recorded on Genesys 10 UV spectrophotometer. Specific optical rotation was determined on Jasco P-2000 polarimeter instrument conditioning with standard compound Fmoc-Ser(tBu)-OH (CAS: 71989-33-8) $[\alpha]_D^{20}$ + 25 (c = 0.1, EtOAc). LC-MS analysis was conducted on Waters Alliance 2690 LC system (C_{18} column, 3 x 75 mm) coupled with Waters ACQUITY QDa mass detector. LC conditions: Solution A (H_2O, 0.05 % Formic acid), Solution B (MeCN). Gradient as follows: 0 min 95 % A and 5 % B; 0-5 min, 5 % A and 95 % B; 5-6 min, 5 % A and 95 % B; 6-7 min, 95 % A and 5 % B. Injection volume was 10 μL. The detection was done at 254 nm. MS detection: ESI positive mode. HRMS analysis was performed Brucker Daltonics microTOF II spectrometer equipped with an orthogonal electrospray (ESI) interface. Calibration was performed using a solution of 10 mM formiate. Sample solutions were introduced into the spectrometer source with a syringe pump (Harvard type 55 111) with a flow rate of 4 μL.min^{-1}

Production and purification of YgbP. pQE-31-ygbP vector containing *E.coli ygbP* gene cloned between KpnI and HindIII restriction sites of pQE-31 vector was used to transform *E.coli* M15 [pREP4], yielding *E.coli* M15 stain [pREP4, pQE31-ygbP][1]. *E. coli* M15 [pREP4, pQE31-ygbP] was grown at 37°C on LB medium (2 x 500 mL) containing ampicillin (100 µg/mL) and kanamycin (25 µg/mL) to 0.6 OD_{600}. Induction was performed with IPTG (100 µM) for 4 h at 37°C. Cells were harvested by centrifugation (7000 x *g*, 10 min, 4°C) and kept at -80°C. *E.coli* M15 [pREP4, pQE31-ygbP] cells (4.92 g) were suspended in 50 mM Tris-HCl buffer (20 mL, pH=8) and sonicated (8 x 30 s with 1 min cooling). After centrifugation (15000 x *g*, 15 min, 4°C), the supernatant was collected and loaded onto a Ni-NTA column (Qiagen, 1.2 x 7 cm column), equilibrated with 50 mM Tris-HCl pH=8. The resin was first washed with 50 mM Tris-HCl pH=8, containing 20 mM imidazole solution (20 mL). The His_6-YgbP was eluted using a linear gradient of imidazole (20-150 mM imidazole, 200 mL, 1 mL min^{-1}) in 50 mM Tris-HCl pH=8 yielding YgbP (65 mg) after pooling the purest fractions (purity more than 90% judged by SDS-polyacrylamide gel electrophoresis). After concentration on Amicon Ultra 15 YM-30 (Millipore), the resulting YgbP solution (3 mL, 17.5 mg mL^{-1}) was divided in aliquots and stored at -80°C. Protein concentration was measured by the method of Bradford using Bovine Serum Albumin as standard.

Preparation of the dye reagent. Malachite green solution was prepared by adding concentrated H_2SO_4 (60 mL) slowly to H_2O (300 mL) and after cooling, the malachite green powder (400 mg) was added to the solution[2]. On the day of the experiment, ammonium molybdate (188 mg) was dissolved in H_2O (2.5 mL) and added to the previously prepared malachite green solution (10 mL) after which tween 20 (200 μL) was added. The solution was mixed until homogenous.

Synthesis of compounds 11-23.

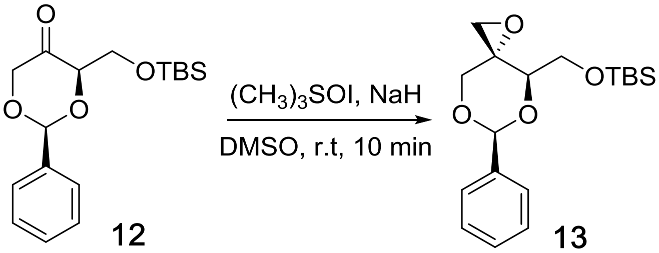

(2*S*,4*R*,5*S*)-cis, trans-4-(tert-butyldimethylsilyloxymethyl)-5, 5-oxymethylene-2-phenyl-1, 3-dioxane (13). To a solution of trimethylsulfoxonium iodide (1.1 eq., 793 mg, 3.61 mmol) in dry DMSO (11 mL) was added pre-crushed NaH (1.1 eq., 144 mg, 3.61 mmol) and the mixture was stirred for 5 minutes. Ketone[3] **12** (1 eq., 1057 mg, 3.28 mmol) in THF (2 mL) was added dropwise to the mixture that was further stirred at room temperature for 10 min. The reaction was quenched by slow addition of water (10 mL), extracted with DCM (5 x 50 mL) and the combined organic layers were dried over $MgSO_4$ before evaporation. The residue was purified by flash chromatography (EtOAc / cHexane : 1 / 9) to give epoxide **13** (582 mg, 53 %) as a colorless oil. Spectral analysis were in agreement with the literature report.[3]

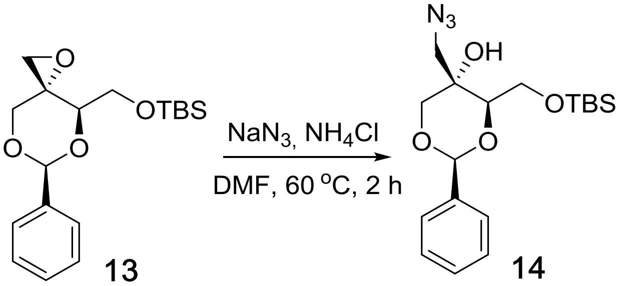

(2*S*,4*R*,5*S*)-5-(azidomethyl)-4-(((tert-butyldimethylsilyl)oxy)methyl)-2-phenyl-1,3-dioxan-5-ol (**14**). To a solution of epoxide **13** (1 eq, 150 mg, 0.446 mmol) in DMF (2.5 mL), NH_4Cl (1.5 eq., 35.8 mg, 0.669 mmol) and NaN_3 (4 eq., 115 mg, 1.78 mmol) were added and the mixture was stirred at 60 °C for 2 h. Water (5 mL) was added before the mixture was extracted with EtOAc (4 x 15 mL). Combined organic layers were washed with H_2O (15 mL), brine (15 mL) then dried over $MgSO_4$ and evaporated. The residue was purified by flash chromatography (EtOAc : cHexane, 2 : 8) to give compound **14** (130 mg, 77 %) as a colorless oil. R_f = 0.48 (EtOAc : cHexane, 2 : 8), $[\alpha]_D^{20}$ + 9.8 (c = 0.2, MeOH), **^{1}H NMR** (400 MHz, $CDCl_3$) δ (ppm): 7.44 (m, 2H), 7.36 (m, 3H), 5.55 (s, 1H), 4.32 (d, *J* = 11.2 Hz, 1H), 4.02 – 3.84 (m, 3H), 3.80 – 3.75 (m, 2H), 3.67 (dd, *J* = 12.8, 0.9 Hz, 1H), 3.61 (dd, *J* = 11.2, 1.1 Hz, 1H), 0.93 (s, 9H), 0.14 (s, 3H), 0.13 (s, 3H). **^{13}C NMR** (101 MHz, $CDCl_3$) δ (ppm): 137.1, 129.2, 128.3, 126.1, 102.2, 79.7, 72.0, 69.0, 62.8, 53.1, 25.8, 18.1, -5.6, -5.7. **IR** (neat) ν (cm^{-1}) 3479 (OH), 2953, 2857, 2101 (N_3), 1255. **MS (ESI+)** *m/z*: 352.19 [M-N_2], 402.18 [M+Na].

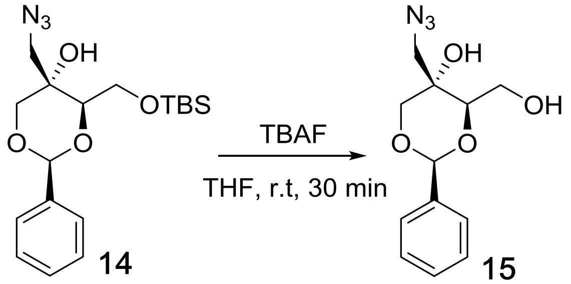

(2*S*,4*R*,5*S*)-5-(azidomethyl)-4-(hydroxymethyl)-2-phenyl-1,3-dioxan-5-ol (**15)**. To a solution of azido alcohol **14** (1 eq., 85 mg, 0.224 mmol) in anhydrous THF (4 mL) at 0 °C, TBAF (1.05 eq., 1 M, 0.235 mL, 0.235 mmol) was added dropwise and the mixture was stirred at room temperature for 30 minutes. Water (5 mL) was added and the product was extracted with ether (4 x 10 mL). Combined organic layers were dried over MgSO4 and evaporated to give compound **15** (55 mg, 93 %) as a colorless oil. R_f = 0.33 (EtOAc : cHexane, 4 : 6), $[\alpha]_D^{20}$ - 8 (c = 0.2, MeOH). **^{1}H NMR** (500 MHz, $CDCl_3$) δ (ppm): 7.46 (m, 2H), 7.39 (m, 3H), 5.55 (s, 1H), 4.24 (d, *J* = 11.1 Hz, 1H), 3.93 – 3.82 (m, 4H), 3.71 (dd, *J* = 12.6, 1.0 Hz, 1H), 3.56 (dd, *J* = 11.1, 1.2 Hz, 1H). **^{13}C NMR** (126 MHz, $CDCl_3$) δ (ppm): 136.9, 129.51, 128.4, 126.1, 102.1, 81.9, 72.2, 67.9, 60.7, 52.8. **IR** (neat) ν (cm^{-1}) 3379 (OH), 2929, 2868, 2105 (N_3). **MS (ESI+)** *m/z*: 238.05 [M-N_2 +H].

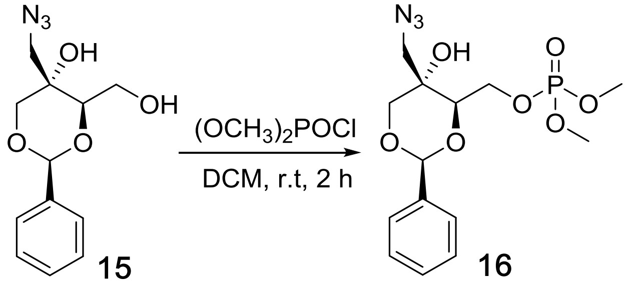

((2*S*,4*R*,5*S*)-5-(azidomethyl)-5-hydroxy-2-phenyl-1,3-dioxan-4-yl)methyl dimethyl phosphate (**16)**. To a solution of alcohol **15** (1 eq., 453 mg, 1.71 mmol) in anhydrous DCM (2.2 mL) at 0 °C, DMAP (1.5 eq., 312 mg, 2.56 mmol) was added followed by dropwise addition of dimethyl chlorophosphate (1.5 eq., 370 mg, 276 µL, 2.56 mmol). The reaction was stirred for 2 hours (monitored by TLC, EtOAc : cHexane, 5 : 5). After completion, the mixture was evaporated and the product purified by chromatography (EtOAc : cHexane 5 : 5 to 7 : 3) to yield **16** (574 mg, 90 %) as a colorless oil. R_f = 0.4 (EtOAc : cHexane, 5 : 5), $[\alpha]_D^{20}$ +10.16 (c = 0.13, MeOH) **^{1}H NMR** (400 MHz, $CDCl_3$) δ (ppm): 7.47 (m, 2H), 7.37 (m, 3H), 5.57 (s, 1H), 4.26 – 4.15 (m, 2H), 4.09 (dd, *J* = 7.3, 3.1 Hz, 1H), 3.89 (d, *J* = 12.5 Hz, 1H), 3.76 (d, *J* = 11.1 Hz, 3H), 3.75 (d, *J* = 11.1 Hz, 3H), 3.66 (d, *J* = 12.5 Hz, 1H), 3.62 (d, *J* = 11.1 Hz, 1H), 2.89 (s, 1H). **^{13}C NMR** (101 MHz, $CDCl_3$) δ (ppm): 136.9, 129.2, 128.3, 126.1, 102.1, 82.0 (d, J_{CP} = 6.5 Hz), 72.6, 67.4, 65.4 (d, J_{CP} = 5.4 Hz), 54.6 (d, J_{CP} = 5.9 Hz), 54.5 (d, J_{CP} = 6.0 Hz), 52.7. **^{31}P NMR** (162 MHz, $CDCl_3$) δ (ppm) 1.31. **IR** (neat) ν (cm^{-1}) 3310 (OH), 2950, 2847, 2100 (N_3), 1450, 1254 (P=O), 1012. **MS (ESI+)** *m/z*: 374.11 [M+H].

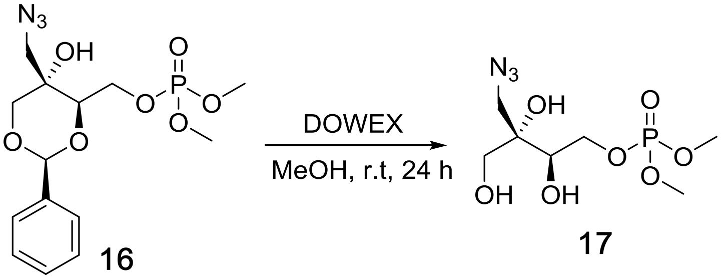

16 17

(2*R*,3*S*)-4-azido-2,3-dihydroxy-3-(hydroxymethyl)butyl dimethyl phosphate (17). To a solution of phosphodimethyl ester **16** (1 eq., 90 mg, 0.241 mmol) in MeOH (1.92 mL) and water (0.3 mL) was added DOWEX 50WX4-200 (50 mg). The suspension was stirred for 24 h. The mixture was then filtered through a glass filter and purified by flash chromatography (MeOH : DCM, 1 : 9) to give compound **17** (30 mg, 44 %) as a colorless oil. R_f = 0.38 (MeOH : DCM, 5 : 5), $[\alpha]_D^{20}$ +2.8 (c = 0.3, MeOH). **^{1}H NMR** (500 MHz, $CDCl_3$) δ (ppm): 4.37 (td, *J* = 11.6, 2.6 Hz, 1H), 4.18 (m, 1H), 3.98 (m, 1H), 3.86 (d, *J* = 4.2 Hz, 1H), 3.82 (d, *J* = 11.2 Hz, 3H), 3.81 (d, *J* = 11.2 Hz, 3H), 3.73 (dd, *J* = 11.4, 5.8 Hz, 1H), 3.66 (dd, *J* = 11.4, 5.8 Hz, 1H), 3.52 and 3.47 (2d, *J* = 12.6 Hz, 2H), 3.03 (s, 1H), 2.37 (t, *J* = 5.9 Hz, 1 H). **^{13}C NMR** (101 MHz, $CDCl_3$) δ (ppm): 75.2, 72.5 (d, J_{CP} = 4.6 Hz), 69.2 (d, J_{CP} = 5.8 Hz), 64.2, 54.8 (d, J_{CP} = 6.0 Hz), 53.2. **^{31}P NMR** (162 MHz, $CDCl_3$) δ (ppm) 2.83. **IR** (neat) ν (cm^{-1}) 3350 (OH), 2099 (N_3), 1445, 1241 (P=O), 1009, 849. **MS (ESI+)** *m/z*: 308.06 [M+Na].

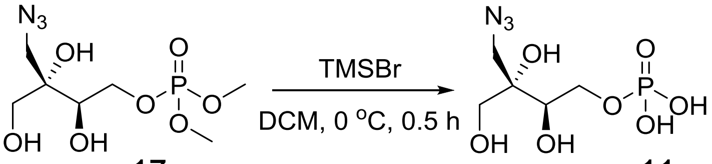

17 11

(2*R*,3*S*)-4-azido-2,3-dihydroxy-3-(hydroxymethyl)butyl phosphate (11). To a solution of phosphodimethyl ester **17** (1 eq., 30 mg, 0.105 mmol) in DCM (2 mL) was added bromotrimethylsilane (6 eq., 96.6 mg, 83.3 µL, 0.631 mmol) in DCM dropwise at 0 °C and stirred for 0.5 h at 0 °C then 0.5 h at room temperature. Solvent was evaporated and water (3 mL) was added and the mixture was further stirred 1 h at room temperature. Water was removed by lyophilization and the residue was purified by flash chromatography on silica gel (Isopropanol : NH_4OH (28%) : H_2O, 6 : 3 : 0.5) to give MEPN$_3$ **11** (7.3 mg, 18 %) as a white solid (di-ammonium salt). R_f = 0.13 (Isopropanol : NH_4OH (28%) : H_2O, 6 : 3 : 0.5), $[\alpha]_D^{20}$ +4.5 (c = 0.1, MeOH) **^{1}H NMR** (500 MHz, D_2O) δ (ppm): 4.04 – 3.99 (m, 1H), 3.90 – 3.85 (m, 2H), 3.67 (d, *J* = 12.0 Hz, 1H), 3.64 (d, *J* = 12.0 Hz, 1H), 3.59 (d, *J* = 13.0 Hz, 1H), 3.51 (d, *J* = 13.0 Hz, 1H). **^{13}C NMR** (126 MHz, D_2O) δ (ppm): 76.12, 71.75 (d, J_{CP} = 6.9 Hz), 64.76 (d, J_{CP} = 4.8 Hz), 62.24, 52.70. **^{31}P NMR** (162 MHz, D_2O) δ (ppm) 2.80. **IR** (neat) ν (cm^{-1}) 3321 (OH), 2109 (N_3), 1423, 1256 (P=O). **HRMS (ESI-)** *m/z*: calculated for 256.0340 [M-H], found 256.0357.

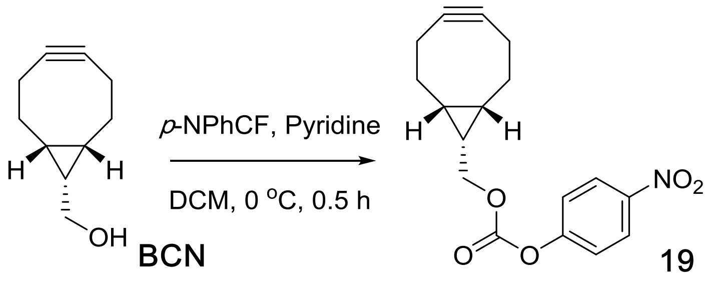

((1*R*,8*S*,9*S*)-bicyclo[6.1.0]non-4-yn-9-yl)methyl (4-nitrophenyl) carbonate (19) was synthesized according to the literature report[4,5] starting from *((1R,8S,9S)-bicyclo[6.1.0]non-4-yn-9-yl)methanol* (BCN).

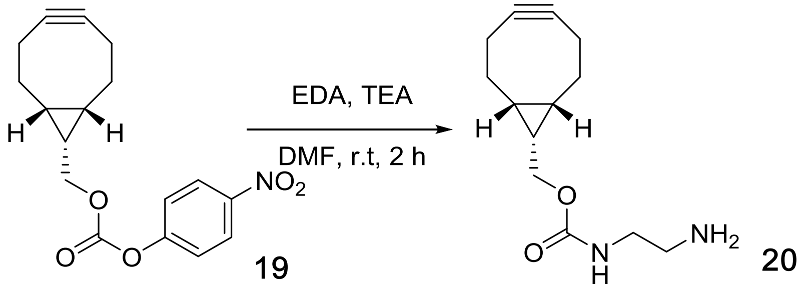

((1*R*,8*S*,9*S*)-bicyclo[6.1.0]non-4-yn-9-yl)methyl (2-aminoethyl)carbamate (20) was prepared according to the literature report[6] starting from compound **19**.

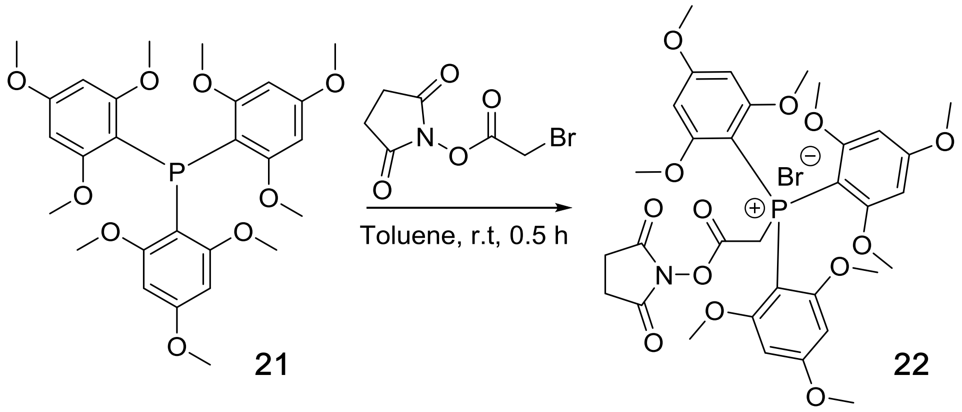

(2-((2,5-Dioxo-1-pyrrolidinyl)oxy)-2-oxoethyl)(tris(2,4,6-trimethoxyphenyl))phosphonium bromide (22) was prepared according to the literature report[7] starting from tris(2,4,6-trimethoxyphenyl)phosphine[8] **21** and bromoacetic acid N-hydroxysuccinimide ester.

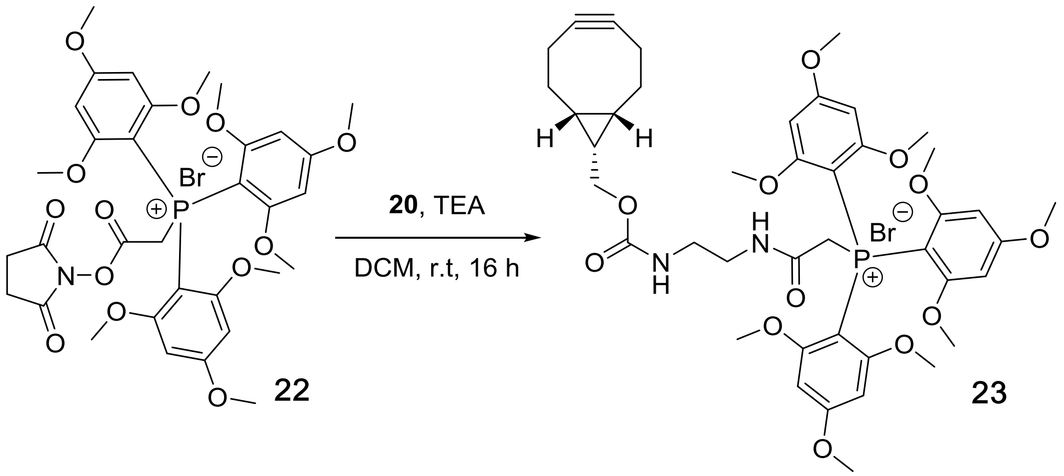

(2-((2-(((((1*R*,8*S*,9*S*)-bicyclo[6.1.0]non-4-yn-9-yl)methoxy)carbonyl)amino)ethyl)amino)-2-oxoethyl)tris(2,4,6-trimethoxyphenyl) phosphonium bromide (23). To a solution of **20** (1 eq., 60 mg, 0.254 mmol) in DCM (3 mL) was added TEA (27 mg, 0.0371 mL, 0.267 mmol) followed by addition of compound **22** (1 eq., 175 mg, 0.254 mmol). The reaction mixture was stirred at room temperature for 16 h. The solvent was removed under reduced pressure and the residue was purified by flash chromatography (DCM : cHexane : MeOH, 4 : 5 :1) to give compound **23** (170 mg, 83 %) as a white solid. R_f = 0.35 (DCM : cHexane : MeOH, 4 : 5 :1), **^{1}H NMR** (400 MHz, $CDCl_3$) δ (ppm): 6.04 (d, J_{HP} = 4.8 Hz, 6H), 4.55 (d, J_{HP} = 15.2 Hz, 2H), 4.09 (d, J = 8.0 Hz, 2H), 3.82 (s, 9H), 3.65 (s, 18H), 3.35 - 3.29 (m, 2H), 3.28 – 3.23 (m, 2H), 2.29 – 2.16 (m, 6H), 1.75 (s, 2H), 1.61 – 1.51 (m, 2H), 1.38 – 1.29 (m, 1H), 0.93 – 0.86 (m, 2H). **^{13}C NMR** (101 MHz, $CDCl_3$) δ (ppm): 166.38 (d, J_{CP} = 3.4 Hz), 165.32 (d, J_{CP} = 1.7 Hz), 163.86 (d, J_{CP} = 1.1 Hz), 157.18, 99.07, 93.10 (d, J_{CP} = 109.4 Hz), 91.00 (d, J_{CP} = 7.3 Hz), 62.25, 56.23, 55.58, 40.56, 40.33, 34.61 (d, J_{CP} = 68.9 Hz) 29.26, 21.61, 20.25, 18.11. **^{31}P NMR** (162 MHz, $CDCl_3$) δ (ppm) -1.19. **IR** (neat) ν (cm^{-1}) 3216 (N-H), 3004, 2941, 2843 (C-H), 1592 (C=O), 1230 (C-O). **MS (ESI+)** *m/z*: 809.75 [M-Br].

Substrate inhibition

The initial rates versus the concentration of the substrate (S) were plotted.

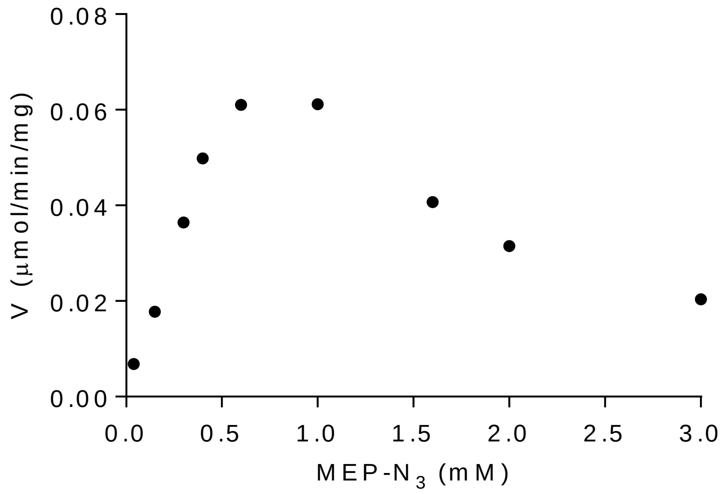

Figure S1. Substrate inhibition of YgbP by MEPN$_3$ **11.** *Conditions:* $MgCl_2$ (1 mM), MEP (0.25 mM), CTP (0.2 mM), DTT (1 mM), Inorganic pyrophosphatase (133 mU/mL), *E.coli* IspD (42 µg) in 400 µL Tris HCl (50 mM, pH = 8).

Bisubstrate kinetic analysis of YgbP.

The kinetic sequential mechanism for an enzyme (E) involving two substrates (A and B) and producing two products (P and Q) is given in Figure S2. K_A represents the dissociation constant of EB complex for A, K_B the dissociation constant of EA complex for B, K_{iB} the Michaelis constant of E for B, K_{iA} the Michaelis constant of E for A. The initial velocity (v) for this Bi Bi sequential kinetic mechanism can be fitted to the equations (1) (when substrate A is varied) and (2) (when substrate B is varied) where V_{max} is the maximum reaction velocity rate[9,10].

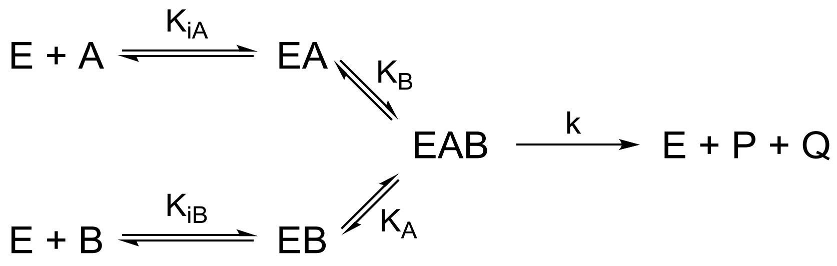

Figure S2. Kinetic sequential mechanism for reactions involving two substrates

$$v = \frac{V_{max}[A][B]}{K_{iA}K_B + K_B[A] + K_A[B] + [A][B]} \qquad (1)$$

$$v = \frac{V_{max}[A][B]}{K_{iB}K_B + K_B[A] + K_A[B] + [A][B]} \qquad (2)$$

The rate equation (1) and (2) for sequential mechanism can be expressed in double reciprocal form as shown in equation (3) (when substrate A is varied) and (4) (when substrate B is varied).

$$\frac{1}{v} = \frac{1}{V_{max}}\left(1 + \frac{K_A}{[A]} + \frac{K_B}{[B]} + \frac{K_{iA}K_B}{[A][B]}\right) \qquad (3) \qquad \frac{1}{v} = \frac{1}{V_{max}}\left(1 + \frac{K_A}{[A]} + \frac{K_B}{[B]} + \frac{K_{iB}K_A}{[A][B]}\right) \qquad (4)$$

Equation (3) can be expressed as equation (5) if 1/*v* is plotted as a primary plot against 1/[A] when substrate A is varied. Similarly equation (6) can be derived from equation (4) if 1/*v* is plotted as a primary plot against 1/[B] when B is the varied substrate.

$$\frac{1}{v} = \frac{1}{V_{max}}\left(K_A + \frac{K_{iA}K_B}{[B]}\right)\frac{1}{[A]} + \frac{1}{V_{max}}\left(1 + \frac{K_B}{[B]}\right) \qquad (5)$$

$$\frac{1}{v} = \frac{1}{V_{max}}\left(K_B + \frac{K_{iB}K_A}{[A]}\right)\frac{1}{[B]} + \frac{1}{V_{max}}\left(1 + \frac{K_A}{[A]}\right) \qquad (6)$$

If slope values from the primary plot are plotted against 1/[B] as a secondary plot when substrate A is varied, equation (7) is derived from equation (5). In the similar manner, equation (8) can be deduced from equation (6) when substrate B is varied if slope values are plotted against 1/[A].

$$\frac{1}{v} = \left(\frac{K_{iA}K_B}{V_{max}}\right)\frac{1}{[B]} + \frac{K_A}{V_{max}} \qquad (7) \qquad \frac{1}{v} = \left(\frac{K_{iB}K_A}{V_{max}}\right)\frac{1}{[A]} + \frac{K_B}{V_{max}} \qquad (8)$$

On the other hand, if 1/*v* axis intercept values from the primary plot are plotted against 1/[B] as a secondary plot when substrate A is varied, then equation (9) is obtained from equation (5). Equation (10) can be formed from equation (6) when substrate B is varied if intercept values are plotted against 1/[A].

$$\frac{1}{v} = \left(\frac{K_B}{V_{max}}\right)\frac{1}{[B]} + \frac{1}{V_{max}} \qquad (9) \qquad \frac{1}{v} = \left(\frac{K_A}{V_{max}}\right)\frac{1}{[A]} + \frac{1}{V_{max}} \qquad (10)$$

From secondary plots of intercepts (equation 9 and 10), V_{max}, K_B and K_A values were deduced. Using these values, K_{iA} and K_{iB} values were determined from secondary plots of slopes (equation 7 and 8). The data were plotted using GraphPad Prism 7.

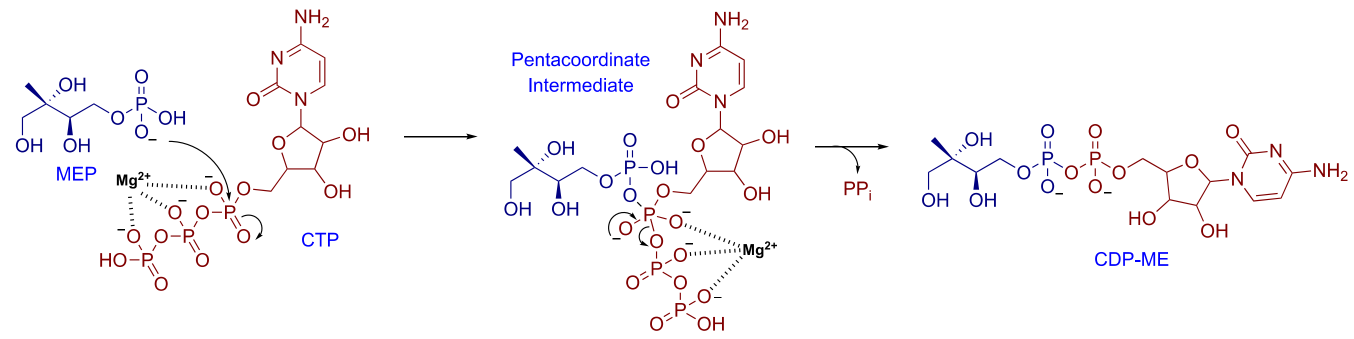

Figure S3. YgbP catalysed reaction mechanism[11]

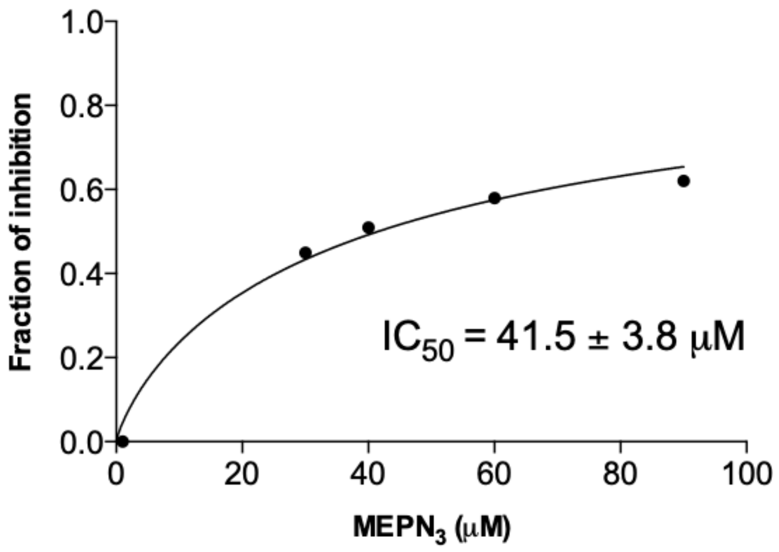

Figure S4. IC_{50} determination of $MEPN_3$. *Conditions*: $MgCl_2$ (1 mM), MEP (0.25 mM), DTT (1 mM), CTP (0.2 mM), Inorganic pyrophosphatase (133 mU/mL), *E.coli* IspD – 0.065 µg in 400 µL Tris HCl (50 mM, pH = 8)

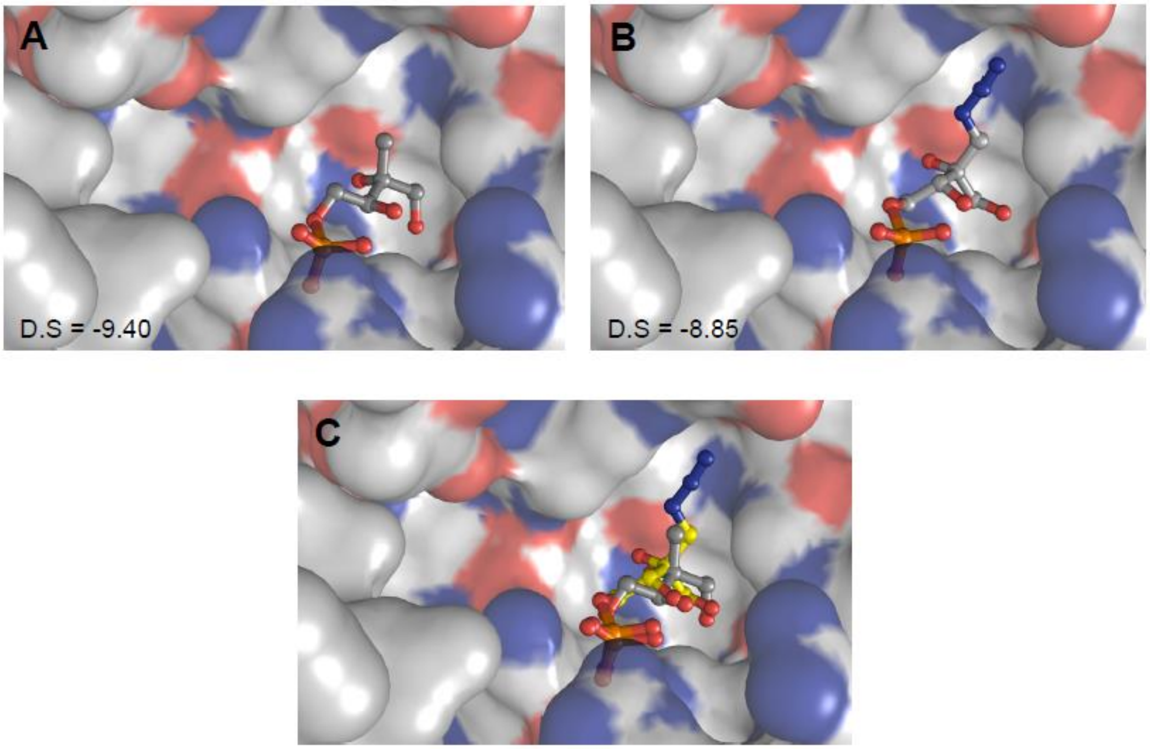

Figure S5. In silico docking results using MEP. Docking experiments were performed with the X-ray structure of *E. coli* YgbP: CTP complex (PDB ID: 1I52). The best docking poses and their corresponding docking scores (D.S) are reported. (A) and (B) MEP and respectively $MEPN_3$ docked using a target with an empty binding pocket. (C) Superposition of MEP and $MEPN_3$ deduced from (A) and (B). $MEPN_3$ is depicted with yellow carbon atoms. The compounds were docked using Glide in extra precision (XP) mode and the Glide docking score was used to rank the docking poses.

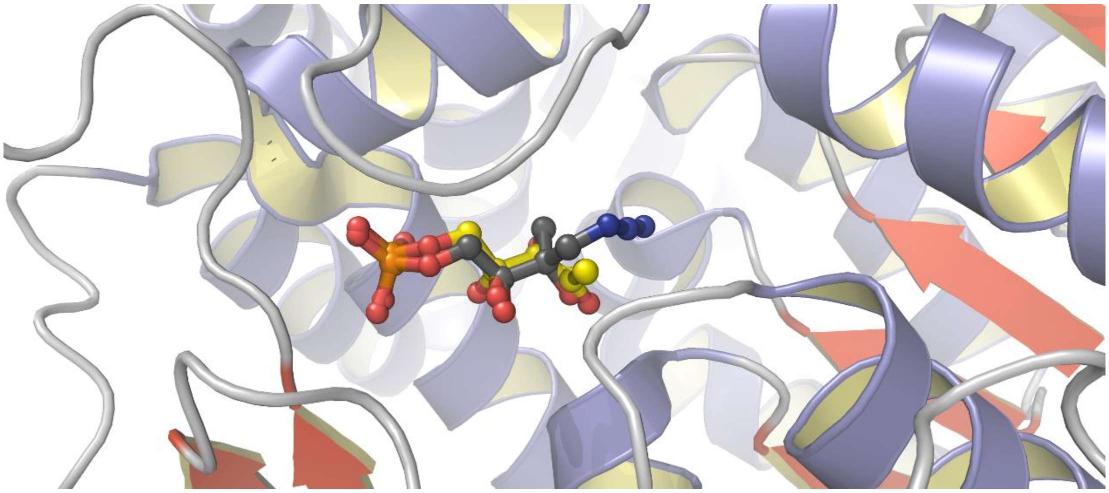

Figure S6. In silico docking of MEPN3 using E. coli DXR. Docking experiments were performed with the X-ray structure of the *E. coli* DXR complex 1-deoxy-D-xylulose-5-phosphate and NADPH (PDB ID: 1Q0Q). Superposition of Glide MEPN$_3$ best pose onto DXR crystallographic structure: 1-deoxy-D-xylulose-5-phosphate from DXR structure is depicted with yellow carbon atoms while the best docking pose (docking score = -9.09) obtain for MEPN$_3$ is depicted with dark gray carbon atoms.

References

1. Bernal, C., Palacin, C., Boronat, A. & Imperial, S. A colorimetric assay for the determination of 4-diphosphocytidyl-2-*C*-methyl-D-erythritol 4-phosphate synthase activity. *Analytical Biochemistry* **337**, 55-61, doi:http://dx.doi.org/10.1016/j.ab.2004.10.011 (2005).
2. Baykov, A. A., Evtushenko, O. A. & Avaeva, S. M. A malachite green procedure for orthophosphate determination and its use in alkaline phosphatase-based enzyme immunoassay. *Analytical Biochemistry* **171**, 266-270, doi:http://dx.doi.org/10.1016/0003-2697(88)90484-8 (1988).
3. Lagisetti, C., Urbansky, M. & Coates, R. M. The Dioxanone Approach to (2S,3R)-2-*C*-Methylerythritol 4-Phosphate and 2,4-Cyclodiphosphate, and Various MEP Analogues. *The Journal of Organic Chemistry* **72**, 9886-9895, doi:10.1021/jo0711900 (2007).
4. Isaacman, M. J., Corigliano, E. M. & Theogarajan, L. S. Stealth Polymeric Vesicles via Metal-Free Click Coupling. *Biomacromolecules* **14**, 2996-3000 (2013).
5. Ursuegui, S., Recher, M., Krężel, W. & Wagner, A. An in vivo strategy to counteract post-administration anticoagulant activity of azido-Warfarin. *Nature Communications* **8**, 15242, doi:10.1038/ncomms15242 https://www.nature.com/articles/ncomms15242 - supplementary-information (2017).
6. D'Alessandro, P. L. *et al.* Bioorthogonal Probes for the Study of MDM2-p53 Inhibitors in Cells and Development of High-Content Screening Assays for Drug Discovery. *Angew Chem Int Edit* **55**, 16026-16030 (2016).
7. Lee, P. J., Chen, W. B. & Gebler, J. C. Qualitative and quantitative analysis of small amine molecules by MALDI-TOF mass spectrometry through charge derivatization. *Analytical Chemistry* **76**, 4888-4893 (2004).
8. Wada, M. & Higashizaki, S. A highly basic triphenylphosphine, [2,4,6-(MeO)3C6H2]3P. *Chem Commun*, 482-483 (1984).
9. Dalziel, K. Initial steady state velocities in the evaluation of enzyme-coenzyme-substrate reaction mechanisms. *Acta Chemica Scandinavica* **11**, 1706-1723 (1957).
10. Purich, D. L. *Enzyme Kinetics: Catalysis & Control, A Reference of Theory and Best-Practice Methods*. (Academic Press, Elsevier, 2010).
11. Richard, S. B. *et al.* Structure of 4-diphosphocytidyl-2-C-methylerythritol synthetase involved in mevalonate-independent isoprenoid biosynthesis. *Nat Struct Biol* **8**, 641-648, doi:Doi 10.1038/89691 (2001).
